# The First Report of Multiple, Bilateral Axillary Epidermal Inclusion Cysts

**DOI:** 10.7759/cureus.55640

**Published:** 2024-03-06

**Authors:** Nathaniel B Hunter, Morgan Rousseau, Emelie E Nelson, Rashid M Rashid

**Affiliations:** 1 Dermatology, University of Texas Health Science Center at Houston, Houston, USA; 2 Internal Medicine, University of Texas Health Science Center at Houston, Houston, USA; 3 Dermatology, Mosaic Dermatology, Houston, USA

**Keywords:** cyst, axillary cyst, dermoid cyst, general dermatology, epidermal inclusion cysts, epidermal inclusion cyst

## Abstract

Epidermal inclusion cysts (EICs) are benign masses that often develop on the face, scalp, neck, and back. Typically, EICs occur secondary to acne or obstructed hair follicles. However, the development of multiple EICs is associated with various syndromes and invasive procedures. Despite their relatively benign nature, a small percentage of EICs have been found to undergo malignant transformation. The complete excision of EICs is essential for their definitive treatment because of their ability to rupture, causing pain and infection. We present the first reported case of a patient without a history of acne, axillary surgery, or genetic syndromes who presented with multiple, painless, bilateral axillary EICs.

## Introduction

Epidermal inclusion cysts (EICs) are benign, cutaneous masses that form most commonly on the face, scalp, neck, and back, often secondary to obstructed hair follicles, acne, or trauma [1-3]. Clinically, EICs present as superficial fluctuant nodules, often containing a visible central punctum. Although most EICs are benign, a small percentage of EICs undergo malignant transformation. Furthermore, the development of multiple EICs has been reported in association with various syndromes, medication use, and following surgery [1]. In this case report, we present the first known case of idiopathic multiple, benign EICs of the bilateral axilla, presenting in a 30-year-old male patient. 

This article was previously presented as a poster presentation at the 2023 Texas Dermatological Society Annual Spring Meeting on April 28, 2023.

## Case presentation

**Figure 1:**
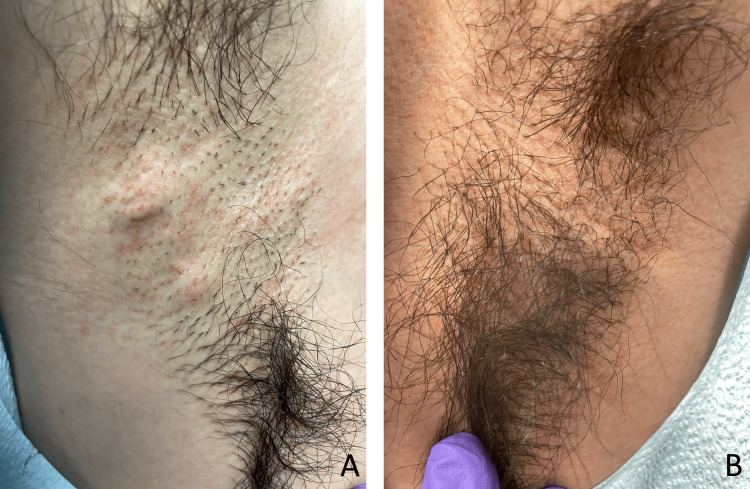
Right axilla with multiple, non-tender nodules (A); left axilla with multiple, non-tender nodules appreciated on palpation (B)

**Figure 2:**
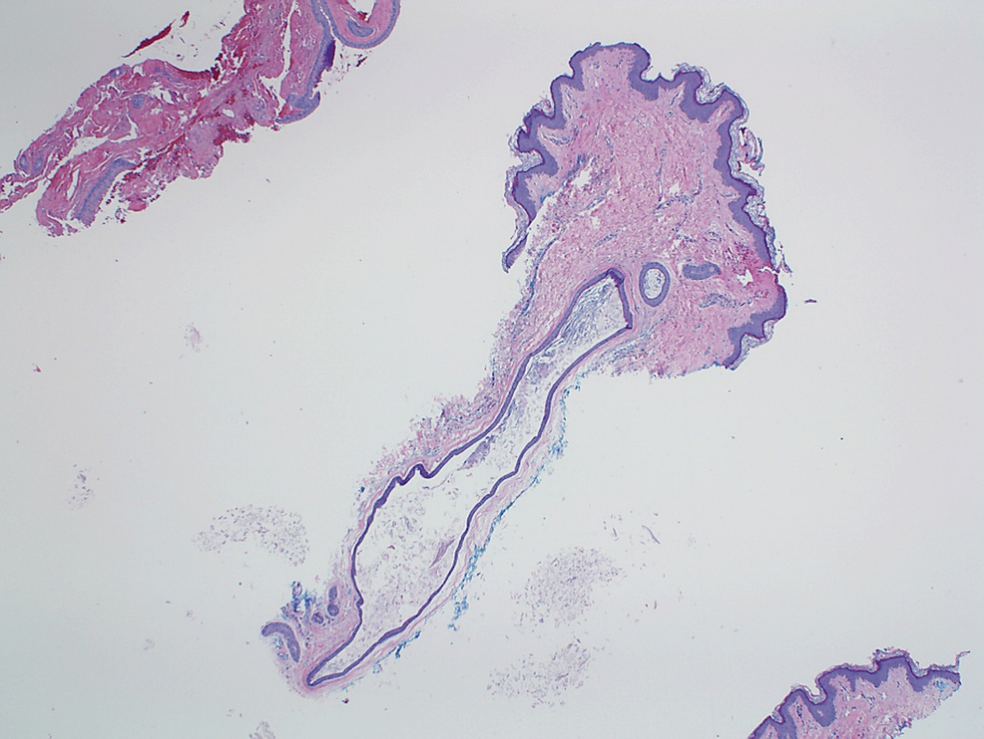
A biopsy demonstrating a cyst lined by stratified squamous epithelium and a keratin-containing cavity (H&E, original magnification 40x)

A 30-year-old male patient presented to the dermatologist with multiple, painless bilateral axillary cysts that had remained unchanged since he was a teenager. He reports feeling increasingly self-conscious about the cysts, especially while at the beach or pool. The patient had no significant past medical history and reported no history of acne, axillary surgery, or genetic syndromes. Physical examination revealed at least five non-tender cysts without central puncta in each axilla that ranged in size from 3 mm to 40 mm (Figure 1). While the presentation of the cysts was subtle, they were readily appreciated upon palpation of the axillae. The patient's axillary cysts were biopsied, and histological results were consistent with epidermal inclusion cysts (Figure 2). Ultimately, the patient declined further treatment or follow-up.

## Discussion

Epidermal inclusion cysts represent the most common benign cutaneous mass and can occur in any anatomic region [1]. EICs present as fluctuant nodules just beneath the skin's surface, and can be painful or painless. Patients typically first notice EICs as a freely movable mass with a visible central punctum. These cysts are often present in adults between the ages of 20-40; however, they can also occur in children as milia. EICs demonstrate a two-to-one male-to-female predominance [1]. 

While EICs most commonly arise sporadically, the presence of multiple EICs has been associated with various syndromes, medication use, and surgery. The predominant anatomic location of EICs varies among the syndromes described. For example, the EICs in Gardner syndrome show a predilection for the extremities [1,4]. In contrast, the EICs in Favre-Racouchot syndrome favor the periocular and nasal regions [5]. The EICs in Gorlin syndrome present in any anatomic region but with two distinct types of cysts: EICs and keratocysts [6]. If a patient presents with lesions in the characteristic syndromic distributions, work-up for a genetic etiology should be considered. BRAF inhibitors can also cause EICs in unusual locations, such as the facial region [1]. Additionally, one case of multiple axillary EICs has been reported postoperatively [7].

EICs demonstrate several characteristic physical exam findings that can aid in their diagnosis. Most EICs are freely movable and flesh-colored; however, they can vary in color depending on secondary infection or rupture [1,8]. EICs will often develop in regions with hair growth and a history of inflammation, irritation, or trauma to the area [1]. However, giant EICs, greater than 5 cm in diameter, often present without a visible punctum, can develop in areas that lack hair growth and display ulcerating features [9]. EICs have the potential to grow in size over time, although many remain stable and do not progress [1]. Patients presenting with an EIC that has changed or grown rapidly should undergo immediate excision and histologic evaluation due to their ability to undergo malignant transformation [1,10].

Dermoscopy can also be used to inform the diagnosis of epidermal inclusion cysts. Dermoscopic evaluation of EICs reveals white, blue, or yellow homogenous regions with the presence of arborizing vessels and may also reveal a punctum that is not visible without magnification [11]. Clinicians can also distinguish between non-ruptured and ruptured EICs with the use of a dermatoscope. Non-ruptured EICs are more likely to display the pore sign and lack peripheral branching vessels. In contrast, ruptured EICs often display the presence of red lacunae and peripheral branching vessels [8]. 

Finally, biopsy and histologic evaluation can aid in the diagnosis of EICs. Histologically, EICs are characterized by a cyst wall lined by stratified squamous epithelium, a keratin-containing cavity, and a granular layer that consists of keratohyalin granules. Additionally, EICs demonstrate the presence of epidermal components in the dermis, with the cyst wall derived from the infundibulum of the hair follicle. Should the EIC become infected, the examination would reveal the formation of a keratin granuloma, neutrophils, disruption of the stratified squamous cyst wall, acute inflammation, and a prominent giant cell reaction [1].

Multiple axillary EICs must be distinguished from other morphological mimics, including hidradenitis suppurativa, benign familial pemphigus, steatocystoma multiplex, and cutaneous lipomas. Axillary hidradenitis suppurativa typically presents as inflamed, draining, tender, and cyclical nodules, frequently with the presence of extensive scarring. While benign familial pemphigus lesions and steatocystoma multiplex lesions present in similar regions to EICs and also produce an exudate, these lesions are vesicular and bullous or yellow-colored, respectively, vesicles and bullae instead of nodules rather than nodular [12,13]. Cutaneous lipomas can present in any anatomic location but are most commonly solitary lesions that are soft and mobile [14]. A biopsy is often necessary to differentiate clinically similar conditions [1]. 

The definitive treatment for EICs is complete excision of the cyst with its walls intact. Multiple methods of excision have been described in the literature, with elliptical excision and punch biopsy being two of the most common [1,15]. One prospective, randomized study revealed that removal of non-inflamed EICs with punch biopsy resulted in significantly quicker operations and smaller scar sizes while maintaining the same standard of postoperative outcomes and complication rates [15]. Regardless of the method used to excise the cyst, the primary goal is to completely eliminate the cyst with its surrounding wall. Failure to eliminate the cyst's walls can result in recurrence, infection, and poor postoperative outcomes [1,4].

Giant EICs can cause significant pain and discomfort for patients and are more likely to rupture spontaneously. As such, they should be removed to prevent secondary infection or abscess formation [1,4]. Triamcinolone is used as a minimally invasive treatment to help shrink the cyst, especially inflamed or giant cysts, prior to surgery. Care must be taken not to directly puncture the EIC when injecting preoperative triamcinolone or anesthetic. If the cyst is punctured, it will likely rupture, increasing the risk of infection and postoperative complications [1]. 

The impact of dermatologic conditions on psychological well-being and quality of life is widely documented in the literature [16-19]. Chronic skin conditions impact various facets of a patient's life, including sleep quality, emotional and psychological health, social functioning, and overall temper [16]. Patients with chronic skin conditions tend to have significantly higher rates of depression, anxiety, and suicidal ideation [17,18]. The profound impact of dermatologic conditions also impacts the ability to function in a professional setting. Many patients report skipping work altogether or experiencing decreased productivity because of their skin condition [18]. Furthermore, some studies have highlighted the direct correlation between the presence of skin disease and the risk of substance abuse [16]. 

Because chronic skin conditions can profoundly impact psychological health, dermatologists are in a unique position to screen patients for depression, anxiety, and suicidal ideation [17]. Many patients with depression secondary to their skin condition may avoid psychiatric help. Because of this, dermatologists may be the only healthcare professional capable of appropriately screening and assessing for psychological disturbances [17]. Some depression screening questionnaires, such as the patient health questionnaire, have helped identify at-risk patients in the dermatologic setting [17,20]. Not only can dermatologists help screen patients for psychiatric conditions, but they also play a critical role in alleviating these conditions by providing reassurance and addressing the underlying skin condition. 

## Conclusions

This case report represents the first known case of idiopathic multiple, benign EICs of the bilateral axilla. While often idiopathic, multiple EICs also can occur in association with various genetic syndromes, medication use, or postoperatively. Despite the subtle presentation of this patient's EICs, they appeared to impact his self-esteem and psychological well-being. This case further highlights the unique role that dermatologists play in identifying and alleviating psychological distress in patients.
